# Physiological Responses to Prior Verbal Notice Before Passive Postural Change: A Randomized Crossover Exploratory Study of Circulatory and Autonomic Dynamics

**DOI:** 10.3390/healthcare14142157

**Published:** 2026-07-17

**Authors:** Yohei Okawa

**Affiliations:** Graduate School of Nursing, St. Luke’s International University, 10-1 Akashi-cho, Chuo-ku, Tokyo 104-0044, Japan; yohei-tky@umin.co.jp; Tel.: +81-80-548-33933

**Keywords:** prior verbal notice, passive postural change, supine position, sitting position, circulatory dynamics, autonomic nervous system, heart rate variability, near-infrared spectroscopy, nursing care

## Abstract

**Background/Objectives:** Passive postural change from the supine position to sitting requires rapid circulatory and autonomic adjustment. This small-scale exploratory study examined whether prior verbal notice is associated with changes in cerebral and peripheral haemoglobin dynamics and heart rate variability during passive postural change in healthy middle-aged and older men. **Methods:** Ten healthy men completed both experimental conditions in randomized order: passive postural change with prior verbal notice and passive postural change without prior verbal notice. Each condition consisted of 10 min of supine rest, 10 min of sitting with feet on the floor, and 10 min in the supine position after return. Near-infrared spectroscopy was used to assess haemoglobin changes in the left frontal region and right gastrocnemius muscle, and heart rate variability was used to estimate autonomic modulation. **Results:** In both conditions, frontal total haemoglobin and oxygenated haemoglobin decreased shortly after sitting and subsequently tended to recover by approximately 5 min after postural change. The low-frequency/high-frequency (LF/HF) ratio increased earlier in the prior-notice condition, and no adverse events occurred. Because the sample was small, these findings should be interpreted as preliminary within-sample patterns rather than as confirmatory evidence. **Conclusions:** Prior verbal notice may be associated with earlier autonomic adjustment during passive postural change. The findings do not establish clinical benefit or causality. Larger crossover studies that include women, frail older adults, and clinical populations are needed before firm clinical recommendations can be made.

## 1. Introduction

Moving from a supine position to sitting produces rapid caudal redistribution of blood volume and requires compensatory cardiovascular and autonomic adjustment. During passive mobilization, these responses may be especially important because the individual cannot actively control the speed or sequence of movement. If these compensatory mechanisms are insufficient, symptoms of orthostatic intolerance, including dizziness, weakness, or syncope, may occur [1,2,3,4]. Previous studies have shown that sitting without back support and changes in body position can alter autonomic modulation, respiratory mechanics, and circulatory stability [5,6,7,8,9,10,11,12,13].

Heart rate variability (HRV) and near-infrared spectroscopy (NIRS) provide noninvasive methods for examining these responses during postural change. HRV spectral indices are typically used to estimate cardiac autonomic modulation, although HF power and the LF/HF ratio should be interpreted as indirect markers rather than direct measurements of parasympathetic and sympathetic nerve outflow [14,15,16,17,18,19,20,21,22,23]. NIRS can continuously monitor changes in oxygenated, deoxygenated, and total haemoglobin; however, frontal NIRS signals may be influenced by superficial blood flow and therefore require cautious interpretation [9,10,11,12,13,16,17,18,19,24,25].

In routine nursing care, verbal notices before mobilization are often provided to reduce surprise, anxiety, and unnecessary physical tension. Physiologically, such a notice may act as an anticipatory cue, allowing autonomic adjustment to begin before the postural stimulus occurs [26,27,28]. However, few studies have investigated whether prior verbal notice is associated with measurable changes in circulatory dynamics and autonomic responses during passive postural change. To address this gap, the present exploratory study focused specifically on the passive transition from the supine position to sitting, prior verbal notice, cerebral and lower-leg haemoglobin dynamics, and HRV-based autonomic indices.

The objective of this study was to examine whether prior verbal notice before passive postural change is associated with differences in circulatory dynamics and autonomic nervous system activity during the transition from the supine position to sitting in healthy middle-aged and older men. The working hypothesis was that prior verbal notice would be associated with earlier autonomic activation and a smoother recovery pattern in frontal haemoglobin changes after sitting.

## 2. Materials and Methods

### 2.1. Study Design and Participants

This was a randomized, two-condition, crossover repeated-measures exploratory study. Participants were not assigned to parallel intervention or placebo groups. Instead, each participant underwent both conditions: passive postural change with prior verbal notice and passive postural change without prior verbal notice.

Participants were recruited publicly at a university. The sample consisted of 10 healthy men aged 40 years or older (mean age, 56.9 ± 10.4 years) with no abnormal findings on health examinations, no history of syncope, no history of serious illness, and no medication use. Written informed consent was obtained from all participants. The experimental procedures and data collection were conducted by one researcher, who was a registered nurse with clinical and research experience in physiological monitoring.

This study was designed as a feasibility-oriented exploratory investigation, and no a priori sample size or statistical power calculation was performed. The small sample size was determined by the feasibility of repeated passive postural change procedures with simultaneous NIRS and HRV monitoring. This design limits statistical power and external validity and is explicitly acknowledged in the Discussion and Conclusions.

### 2.2. Randomization, Experimental Order, and Carryover Considerations

Each participant completed both conditions in randomized order using a simple two-sequence crossover approach: sequence A, no prior notice followed by prior notice; or sequence B, prior notice followed by no prior notice. The participants were not informed of the assigned order. To reduce expectancy effects, participants were told that they might undergo cycles with prior notice only, cycles without prior notice only, or both types of cycles. No auditory signal was used between experimenters, and experimenter movements were minimized.

### 2.3. Experimental Protocol and Environmental Conditions

Before the measurements were taken, height, weight, resting pulse rate, and blood pressure were recorded. Participants were instructed to remain passive and not perform voluntary movements during postural change. They were allowed to experience the passive postural change once before measurement to ensure safety and procedural understanding.

**Figure 1:**
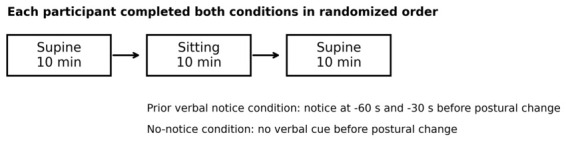
Revised experimental protocol showing the randomized crossover repeated-measures design.

Each condition consisted of 10 min of supine rest, passive transition to sitting with both feet on the floor, 10 min of sitting, and 10 min in the supine position after return (Figure 1). In the prior-notice condition, standardized verbal notices were provided 1 min and 30 s before postural change: “You will soon be raising your body and sitting up, but please do not exert any force”. The timing, volume, and wording of the notices were standardized.

Measurements were conducted in a quiet university laboratory between 10:00 AM and 4:00 PM from July to September. Visual and auditory stimuli other than the prior notice were minimized. Participants were instructed to refrain from strenuous physical exercise and alcohol consumption beginning the day before the experiment. Respiration was not paced, and caffeine intake, meal timing, exact room temperature, and respiratory rate were not strictly standardized or continuously recorded; these factors are acknowledged as possible influences on HRV.

### 2.4. Passive Positional Change

Participants rested horizontally in the supine position on a bed. The headrest was raised to 45 degrees, after which the researcher fully assisted each participant from sitting to a lateral position and then to a seated position on the edge of the bed. The hip, knee, and ankle joints were adjusted so that both soles were in contact with the floor. The back was unsupported while the participants were sitting, and each participant viewed symbols placed directly in front of them on a whiteboard. The time required for each postural change was kept within 30 s.

### 2.5. Outcome Measures

Blood pressure was measured with an Omron digital automatic blood pressure monitor (HEM-759P; OMRON Healthcare Co., Ltd., Kyoto, Japan) attached to the left upper arm. Systolic and diastolic blood pressure were measured 5 min before the positional change and 1 min and 5 min after the start of the positional change. The arm position was adjusted so that the cuff was at heart level in both the supine and sitting positions.

Autonomic nervous system activity was assessed using HRV spectral analysis. Electrocardiogram electrodes were attached to the chest, QRS waves were detected using bipolar lead II, and the analogue signal was transferred to a personal computer using a memory heart rate monitor. Coarse-graining spectral analysis was used to separate the harmonic oscillator components from the fractal components. The low-frequency (LF) component was defined as 0–0.15 Hz, and the high-frequency (HF) component was defined as 0.15–0.5 Hz. HF and LF/HF were interpreted as indirect indices of autonomic modulation, not as direct measures of vagal or sympathetic nerve firing [14,15].

Tissue oxygenation was measured using NIRS (NIRO-200; Hamamatsu Photonics K.K., Hamamatsu, Japan) at wavelengths of 775, 810, and 850 nm. Oxygenated haemoglobin (oxy-Hb), deoxygenated haemoglobin (deoxy-Hb), and total haemoglobin (total-Hb) levels were recorded. The left frontal region and the medial portion of the right gastrocnemius muscle were assessed. NIRS provides continuous monitoring of haemoglobin changes during physiological tasks, but frontal measurements may include extracerebral components such as superficial forehead blood flow [24,25].

### 2.6. Statistical Analysis

Participant characteristics and physiological outcomes are reported as the mean ± standard deviation. Group-level time-course figures include 95% confidence intervals around the mean. Because both condition (prior notice vs. no prior notice) and time (rest, 1 min, and 5 min after sitting) were within-participant factors, the principal outcomes were reanalysed using two-factor repeated-measures analysis of variance (ANOVA), with particular emphasis on the condition × time interaction that directly addresses whether prior verbal notice modifies the physiological response over time. The partial eta squared (ηp^2^) was calculated from the F statistic and its degrees of freedom as an effect-size estimate for each ANOVA effect. Within-condition comparisons were retained as secondary exploratory analyses using one-way repeated-measures ANOVA for normally distributed variables and Friedman tests for nonnormally distributed variables, with Bonferroni correction for multiple comparisons. Exact *p* values are reported when available. Given the very small sample size, all analyses were considered exploratory and hypothesis-generating rather than confirmatory.

### 2.7. Ethical Considerations

The study was conducted in accordance with the Declaration of Helsinki and was approved by the University of Tokai Institutional Review Board. The researchers explained the study in writing, and all the participants provided written informed consent. Participants were informed of the purpose and procedures, their right to withdraw, the handling of data, and privacy protection.

## 3. Results

### 3.1. Participant Characteristics

The participants were 10 healthy men. No adverse events, including syncope, occurred during the measurements, and no participant discontinued the experiment. The mean age was 56.9 ± 10.4 years (Table 1).

### 3.2. Blood Pressure, HRV, and NIRS Outcomes

Table 2 and Table 3 presents group-level physiological values for both conditions. In the two-factor repeated-measures ANOVA, no statistically significant condition × time interaction was observed for log HF (F(2,18) = 0.19, *p* = 0.830, ηp^2^ = 0.020), log LF/HF (F(2,18) = 1.46, *p* = 0.259, ηp^2^ = 0.140), oxy-Hb (F(2,18) = 0.42, *p* = 0.661, ηp^2^ = 0.045), deoxy-Hb (F(2,18) = 0.70, *p* = 0.510, ηp^2^ = 0.072), or total-Hb (F(2,18) = 1.02, *p* = 0.379, ηp^2^ = 0.102). Significant main effects of time were observed for log LF/HF (F(2,18) = 16.87, *p* < 0.001, ηp^2^ = 0.652), deoxy-Hb (F(2,18) = 10.98, *p* < 0.001, ηp^2^ = 0.550), and total-Hb (F(2,18) = 3.93, *p* = 0.038, ηp^2^ = 0.304). These interaction results do not demonstrate that prior verbal notice significantly modified the measured physiological response over time.

**Figure 2:**
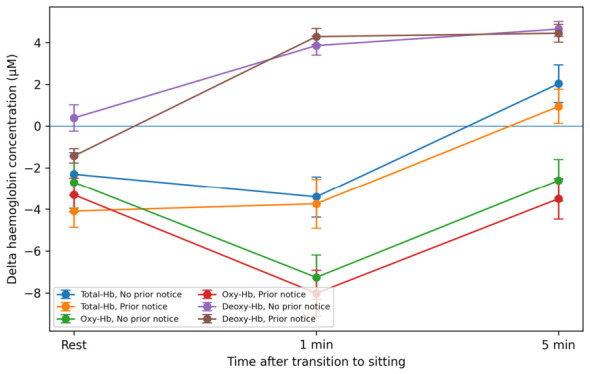
Group-level NIRS time-course values for total-Hb, oxy-Hb, and deoxy-Hb. The error bars indicate 95% confidence intervals around the mean.

Frontal total-Hb and oxy-Hb decreased after sitting in both conditions and tended to recover by 5 min. Deoxy-Hb increased after the postural change, particularly in the lower leg. Group-level NIRS curves with 95% confidence intervals are presented in Figure 2.

**Figure 3:**
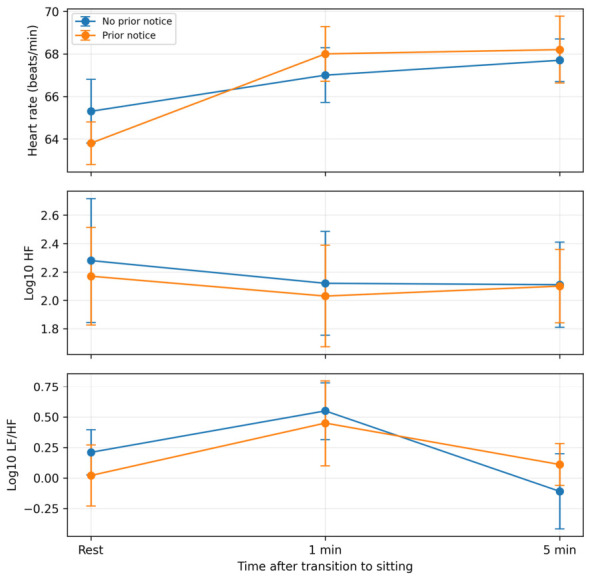
Group-level autonomic and heart rate time course values. The error bars indicate 95% confidence intervals around the mean.

The LF/HF ratio increased after sitting in both conditions. The time-course values for the autonomic indices and heart rate are shown in Figure 3.

## 4. Discussion

This exploratory crossover study identified transient decreases in frontal total-Hb and oxy-Hb after a passive transition from the supine position to sitting, followed by partial recovery by approximately 5 min. Importantly, the reanalysis using two-factor repeated-measures ANOVA did not identify a statistically significant condition × time interaction for the principal HRV or NIRS outcomes. Thus, although the prior-notice condition resulted in a descriptively earlier increase in the LF/HF ratio, the present data do not provide statistical evidence that prior verbal notice modified the physiological response over time. The significant main effects of time for log LF/HF, deoxy-Hb, and total-Hb indicate that physiological responses changed across the postural transition regardless of condition.

These findings are broadly consistent with previous work showing that posture and supported or unsupported sitting can influence autonomic nervous activity. Studies of sitting without back support have reported changes in sympathetic activity and respiratory mechanics during upright positioning [5,6,9,10,11,12,13,29,30,31]. Studies of recumbent positions in older adults and patients with cardiovascular disease have also demonstrated that body position influences cardiac autonomic modulation [3,4,7,8,32,33,34]. The present study extends this literature by focusing on the verbal cue provided immediately before passive postural change rather than on posture alone.

The possible mechanism is that verbal notice may serve as an anticipatory cue [26,27,28]. Anticipatory autonomic adjustment could begin before or at the onset of movement, thereby reducing surprise and allowing the participant to prepare psychologically and physiologically. However, this interpretation remains speculative. The present study measured HRV and NIRS responses but did not measure anxiety, perceived preparedness, respiratory pattern, beat-to-beat blood pressure, or cerebral blood flow velocity. Therefore, causal pathways cannot be established.

Several limitations require emphasis. First, the sample included only 10 healthy men, and no a priori power calculation was performed. Therefore, the study was underpowered for small or moderate effects and cannot support strong clinical inference. Second, the mean age and standard deviation indicate age heterogeneity; participants included both middle-aged and older adults, and age-related autonomic differences may have influenced the results. Third, women, hospitalized patients, frail older adults, and patients with neurological or cardiovascular disorders were not included; therefore, the results cannot be generalized to these groups. Seventh, the original analytical approach emphasized within-condition comparisons, which do not directly test the principal research question of whether prior verbal notice modifies the response over time. Although a two-factor repeated-measures ANOVA was added in response to this concern, the sample of 10 participants provides limited power for interaction effects; therefore, a nonsignificant condition × time interaction should not be interpreted as proof of equivalence or the absence of a potentially meaningful effect. Eighth, the sample was restricted to men to reduce biological heterogeneity in this initial feasibility study, and calf NIRS measurements may be influenced by subcutaneous tissue thickness; consequently, the findings should not be generalized to women without further study.

Fourth, the two experimental conditions were performed consecutively without a formal washout period. Although the order was randomized, carryover, learning, or expectancy effects may have influenced the second condition. Fifth, environmental and behavioural factors relevant to HRV, including respiratory rate, caffeine intake, meal timing, and exact room temperature, were not fully controlled or recorded. Sixth, NIRS provides a useful continuous signal, but frontal measurements may include extracerebral blood flow components, and HRV indices are indirect markers of autonomic modulation rather than direct neural measurements [20,21,22,23].

Future studies should use a larger adequately powered randomized crossover design with a washout period, balanced order allocation, and mixed-effects analysis [35]. They should include women and clinical populations; assess age-stratified responses; record respiratory and environmental variables; and combine physiological measures with patient-centred outcomes such as dizziness, discomfort, anxiety, and perceived preparedness. Such studies are necessary before prior verbal notice can be recommended as a clinically effective intervention for reducing adverse physiological effects during mobilization.

## 5. Conclusions

Passive postural change was accompanied by time-dependent changes in autonomic and haemoglobin-related measures. However, no statistically significant condition × time interaction was detected for the principal HRV or NIRS outcomes, and the present exploratory data do not establish that prior verbal notice modifies the physiological response over time. Larger, adequately powered randomized crossover studies with washout periods and more diverse participants are needed.

## Figures and Tables

**Table 1:** Participant characteristics.

Variable	Unit	Mean	SD
Age	years	56.9	10.4
Height	cm	170.0	6.1
Weight	kg	67.7	9.2
Systolic blood pressure	mmHg	125.9	12.5
Diastolic blood pressure	mmHg	78.3	6.9

**Table 2:** Group-level physiological outcomes by condition and time point (mean ± SD).

Outcome	Condition	Rest	1 min After Sitting	5 min After Sitting
Systolic BP	No prior notice	124.80 ± 12.50	126.30 ± 11.50	125.70 ± 12.80
Systolic BP	Prior notice	127.90 ± 15.00	125.00 ± 9.70	129.70 ± 11.00
Diastolic BP	No prior notice	78.50 ± 8.70	78.20 ± 8.80	81.10 ± 9.60
Diastolic BP	Prior notice	78.50 ± 7.90	78.90 ± 8.30	81.70 ± 8.70
HR	No prior notice	65.30 ± 2.10	67.00 ± 1.80	67.70 ± 1.40
HR	Prior notice	63.80 ± 1.40	68.00 ± 1.80	68.20 ± 2.20
Log HF	No prior notice	2.28 ± 0.61	2.12 ± 0.51	2.11 ± 0.42
Log HF	Prior notice	2.17 ± 0.48	2.03 ± 0.50	2.10 ± 0.36
Log LF/HF	No prior notice	0.21 ± 0.26	0.55 ± 0.33	−0.11 ± 0.43
Log LF/HF	Prior notice	0.02 ± 0.35	0.45 ± 0.49	0.11 ± 0.24
Total-Hb	No prior notice	−2.31 ± 1.45	−3.41 ± 1.36	2.03 ± 1.26
Total-Hb	Prior notice	−4.09 ± 1.09	−3.74 ± 1.65	0.94 ± 1.14
Oxy-Hb	No prior notice	−2.70 ± 1.76	−7.26 ± 1.49	−2.61 ± 1.40
Oxy-Hb	Prior notice	−3.31 ± 1.14	−8.02 ± 1.53	−3.50 ± 1.36
Deoxy-Hb	No prior notice	0.39 ± 0.88	3.85 ± 0.64	4.64 ± 0.50
Deoxy-Hb	Prior notice	−1.43 ± 0.48	4.28 ± 0.55	4.44 ± 0.59

**Table 3:** Key exploratory within-sample contrasts and descriptive effect estimates.

Outcome	Condition	Contrast	Mean Difference	Descriptive SMD	*p* Value
Systolic BP	Prior notice	1 min to 5 min	+4.70	+0.45	*p* < 0.05
Log LF/HF	No prior notice	Rest to 1 min	+0.34	+1.14	*p* < 0.05
Log LF/HF	No prior notice	1 min to 5 min	−0.66	−1.72	*p* < 0.01
Log LF/HF	Prior notice	Rest to 1 min	+0.43	+1.01	*p* < 0.05
Total-Hb	No prior notice	1 min to 5 min	+5.44	+4.15	*p* < 0.05
Deoxy-Hb	No prior notice	Rest to 5 min	+4.25	+5.94	*p* < 0.05
Deoxy-Hb	Prior notice	Rest to 1 min	+5.71	+11.06	*p* < 0.05
Deoxy-Hb	Prior notice	Rest to 5 min	+5.87	+10.91	*p* < 0.05

Note: SMD = standardized mean difference calculated descriptively from summary statistics. Because individual paired-difference standard deviations were not available in the summary table, effect estimates should be interpreted as descriptive rather than confirmatory.

## Data Availability

The data are not publicly available because of privacy and ethical restrictions related to identifiable research materials.
